# RE: The WISDOM Personalized Breast Cancer Screening Trial: Simulation Study to Assess Potential Bias and Analytic Approaches

**DOI:** 10.1093/jncics/pkaa015

**Published:** 2020-02-28

**Authors:** Kimbroe Carter, Frank Castro, Roy Morcos

**Affiliations:** p1 Pathology, Northeast Ohio Medical University, Rootstown, OH, USA; p2 School of Technology, Kent State University Trumbull Campus, Warren, OH, USA; p3 Medical Decision Making Society of Youngstown Ohio, OH, USA; p4 Family and Community Medicine, Northeast Ohio Medical University, Rootstown, OH, USA

In the article *The WISDOM Personalized Breast Cancer Screening Trial: Simulation Study to Assess Potential Bias and Analytical Approaches* by Martin Eklund et al., the authors describe a simulation based on the Women Informed to Screen Depending on Measures of Risk (WISDOM) (1, 2). The intent of this simulation is to investigate sources of ascertainment bias related to WISDOM’s short study time frame and issues surrounding entry and exit into the trial (1). This evaluation is interesting, but the model’s unrealistic assumption of disease existing only at stage IIB or higher distorts outcomes and calls into question the validity of the results.

A primary study objective of WISDOM is to test whether personalized screening is safe, as measured by the noninferiority in the proportion of stage IIB or higher cancers found in the personalized and annual screening arms (2–4). While the modelers are focused on estimating the time to mammogram-detectable stage IIB disease, the opportunity to find cancers at an earlier stage is overlooked. Early-stage disease does not exist in this model.

The purpose of screening is to detect early, asymptomatic disease that is potentially curable (5). Clinically, such cancers could be detected with more frequent screening, thus reducing the proportion of stage IIB or higher disease. With the unrealistic assumption of no early-stage cancers, one would expect similar proportions of stage IIB or higher disease in either study arm, leading one to conclude falsely the noninferiority of the personalized screening strategy.

To properly evaluate screening strategies for breast cancer, a natural history model of this disease should consider growth and development from a size below the detectability threshold of the technologies under investigation. This allows for the incremental benefits of earlier detection with more screening to become apparent. The omission of early-stage disease in this model’s design is fundamentally limiting and masks differences in disease stage between personalized and annual screening strategies.

## Note

The authors have no conflicts of interest to disclose.
